# Artificial Intelligence Detection of Occlusive Myocardial Infarction from Electrocardiograms Interpreted as “Normal” by Conventional Algorithms

**DOI:** 10.3390/jpm15040130

**Published:** 2025-03-28

**Authors:** Shifa R. Karim, Hans C. Helseth, Peter O. Baker, Gabriel A. Keller, H. Pendell Meyers, Robert Herman, Stephen W. Smith

**Affiliations:** 1 Baylor University, Waco, TX 76798, USA; shifa_karim1@baylor.edu; 2 Department of Emergency Medicine, Abbott Northwestern Hospital, Minneapolis, MN 55407, USA; 3 Department of Emergency Medicine, University of Minnesota Medical School, Minneapolis, MN 55455, USA; 4 Hennepin EMS, Hennepin Healthcare, Minneapolis, MN 55415, USA; gabriel.keller@hcmed.org; 5 Department of Emergency Medicine, Carolinas Medical Center, Charlotte, NC 28203, USA; harvey.meyers@atriumhealth.org; 6 Powerful Medical, New York, NY 10011, USA; robert@powerfulmedical.com; 7 Department of Emergency Medicine, Hennepin Healthcare, Minneapolis, MN 55415, USA; 8 Department of Emergency Medicine, University of Minnesota, Minneapolis, MN 55455, USA

**Keywords:** triage, EKG, ECG, electrocardiogram, artificial intelligence, myocardial infarction, occlusion myocardial infarction, acute coronary occlusion

## Abstract

**Background**: Some authors advocate that ECGs with conventional computer algorithm (CCA) interpretations of “normal” need not be immediately reviewed. However, such ECGs may actually manifest findings of acute coronary occlusion myocardial infarction (OMI). We sought to determine if such cases can be detected by artificial intelligence (AI). **Methods**: We studied a retrospective series (2014–2024) of cases with ≥1 pre-angiography ECGs with a proven OMI outcome with a CCA ECG interpretation of “normal”. The OMI outcome was defined as (1) the diagnosis of acute type I MI, (2) an angiographic culprit with intervention, and (3) one of the following, (a) TIMI-0-2 flow, or (b) TIMI-3 or unknown flow, with high peak troponin or new wall abnormality. Each ECG as retrospectively interpreted by the PMcardio OMI AI ECG model. The primary analysis was the performance of AI in diagnosing "OMI" among these CCA “normal” ECGs. **Results**: Forty-two patients with OMI met the inclusion criteria. The first ECG was interpreted as “normal” by the CCA in 88% of cases; AI interpreted 81% as OMI and 86% as abnormal. Of the 78 total ECGs interpreted by the CCA, 73% were diagnosed as “normal”. Of this 73%, AI identified 81% as abnormal and 72% as OMI. **Conclusion**: The Conventional Computer Algorithm may interpret an ECG manifesting OMI as “normal”. AI not only recognized these as abnormal, but in 81% of patients, correctly recognized OMI on the first ECG and recognized 72% of all the CCA “normal” ECGs as OMI. It was rare for AI to diagnose a normal ECG for any OMI patient.

## 1. Introduction

The 12-lead electrocardiogram (ECG) is the primary means of rapidly diagnosing acute coronary occlusion myocardial infarction (ACOMI, shortened to OMI) in patients who have a clinical presentation consistent with acute coronary syndrome (ACS). The importance of diagnosing OMI beyond the STEMI criteria is now very well established in the literature [1,2,3,4,5]. The rapid recognition of OMI is essential for rapid intervention in order to save the myocardium [6]. For patients with chest pain, the guidelines thus recommend that an ECG be recorded and interpreted in the Emergency Department within 10 min. Thus, emergency physicians are frequently interrupted in order to read triage ECGs. However, only a small fraction of patients with chest pain have acute OMI, and so emergency physicians must be interrupted very frequently in order to find that proverbial “needle in a haystack”. It is standard to integrate ECG interpretation algorithms into ECG machines; these conventional algorithms are programmed, instructional, “if-then” algorithms, in contrast to deep neural network artificial intelligence (AI) algorithms. Four recent publications have purported to show that if the conventional computer algorithm (CCA) interpretation of the ECG is “normal”, in contrast to even minor abnormalities such as “nonspecific ST-T abnormality”, the physician need not review the triage ECG because, they contend, there will be no emergency revealed on the ECG [7,8,9,10]. At least one other study contended that physicians’ review of triage ECGs is not cost-effective [11]. Some editorials and letters to editors have disputed this idea [12,13]. These four studies had slightly varying definitions of normal: (1) “Normal sinus rhythm or Normal ECG” or “Normal sinus rhythm with sinus arrhythmia” [7]; (2) “Normal ECG” [8,10]; and (3) “Normal” or “Otherwise normal” ECG [9].

Recent research has shown that there are many subtle ECG findings beyond ST Elevation that are specific for OMI [4,14,15,16]. Furthermore, it has been shown that the CCA interprets some OMI ECGs as completely normal [17]. Over a 10-year period, many ECGs manifesting subtle signs of OMI and proven to be OMI by the patients’ actual clinical outcomes, but interpreted as completely normal by the conventional algorithm, were collected as part of an online blog (Dr. Smith’s ECG Blog) [18]. Artificial intelligence (AI) has been shown to be accurate in detecting OMI that is not STEMI [19,20,21,22]. To our knowledge, only one such deep convolutional neural network model is available for the interpretation of ECG images and has published performance results for the diagnosis of OMI (“Queen of Hearts” PMcardio OMI AI ECG model) [21]. We sought to assess the performance of “Queen of Hearts” on these CCA “normal” ECGs.

## 2. Methods

### 2.1. Study Design

This study was a retrospective case series examining the diagnostic performance of an AI algorithm known as the Queen of Hearts (details below) for the diagnosis of OMI from ECGs misinterpreted as normal by conventional algorithms. All the proven OMI cases that had at least one ECG that Dr. Smith recognized as OMI, but which was interpreted as normal by the CCA and were posted on Dr. Smith’s ECG blog [18] were searched for on the blog and included. Eleven additional cases which met the inclusion criteria outlined below were provided by co-author HH from cases for which he recorded ECGs as a technician in the Minneapolis Heart Institute (MHI) Emergency Department and were collected within the same timeframe and were also recognized as OMI by Dr. Smith. There were numerous other contributors from hospitals across the US over a 10-year period from December 2014 through October 2024, but the vast majority of cases come from either Hennepin County Medical Center or from the MHI or hospitals that refer to the MHI. Any ECGs which were utilized to train the algorithm were excluded. The inclusion criteria included the following: (1) OMI was recognized by Dr. Smith on at least one pre-PCI ECG that was labeled as “normal” by the CCA, and (2) the instance meets the outcome definition of OMI, defined in accordance with previous studies [16,23,24,25,26].

This outcome definition is given as follows:

(1) The diagnosis of acute type 1 MI with rise and/or fall of troponin level; and
(2) Angiographic culprit with intervention; and
(3) One of the following:
  (A) TIMI-0-2 flow;
  (B) TIMI-3 flow or unknown flow with large acute infarct size, as determined by one of the following:
    (i) Very elevated troponin defined as follows:
      (a) Peak high-sensitivity troponin I level > 5000 ng/L;
      (b) Peak 4th generation troponin I > 10 ng/mL;
      (c) Peak high-sensitivity troponin T > 1000 ng/L;
      (d) Peak 4th generation troponin T > 1.0 ng/mL.
    (ii) New regional wall motion abnormality (WMA) on echocardiography if peak troponin levels were not available or were below the very high threshold.

An ECG manifested OMI if the outcome was OMI and Dr. Smith interpreted it as OMI.

The work phase involving the blog posts was conducted using publicly available data and de-identified case descriptions; therefore, IRB approval is not applicable.The cases from the MHI were deemed by the IRB as exempt from IRB approval because they do not represent human subject research.

### 2.2. Data Elements

The blog posts list sex, age, or nearest decade of life; a brief case summary; and a chronological description of events along with high-resolution images of the ECGs obtained at various stages of the patients’ care. All the ECGs recorded prior to angiogram were included for analysis. CCA interpretation as well as the CCA manufacturer were recorded for each ECG when available. CCA interpretation was considered normal if the interpretation listed (1) “Normal ECG”, or (2) “sinus bradycardia/tachycardia, otherwise normal” or (3) “Early repolarization, otherwise normal”. In order to accurately compare the performances of the CCA with the AI system, when there was no CCA interpretation provided on the blog post, but the ECG manifested ST elevation millimeter criteria, we assigned an assumed CCA interpretation of “STEMI”. If an ECG without CCA interpretation provided by the blog post did not meet the millimeter criteria for STEMI, we assigned an interpretation of “unknown”. An interpretation of “normal” was never assumed for any CCA interpretation.

All the ECGs were then reviewed by independent members of the study team for the previously described key elements suggestive of OMI, including hyperacute T-waves, subtle STE (especially when associated with pathological Q-waves or any amount of reciprocal ST depression or reciprocal T-waves, terminal QRS distortion, horizontal ST-segment flattening or down-up T-waves), or any amount of ST depression in V1–V4. The included ECGs were also reviewed by blinded study team members for the STEMI millimeter criteria in accordance with the 4th Universal Definition of MI [27].

### 2.3. Artificial Intelligence Algorithm

The AI algorithm employed herein was version 1 of the Queen of Hearts (QoHs) software powered by the PMcardio OMI AI ECG model, version 2.8 (Powerful Medical; Bratislava, Slovakia). This algorithm first converts any ECG image to digital data, and then employs a deep convolutional neural network (DCNN) model trained to detect OMI from single 12-lead ECG data, blinded to all the other clinical data. In the validation study, the algorithm was twice as sensitive of that of the STEMI criteria for OMI (66% vs. 33%) at a fixed specificity of 97.7% [21]. The current version does not allow for the analysis of serial ECGs, so even the subsequent ECGs from a single patient were interpreted separately without comparison or relation to one another. QoHs reports either “OMI” or “Not OMI” followed by a “high”, “mid”, or “low” confidence rating. The included ECGs were digitally copied from the blog posts and directly uploaded to the software through the Telegram interface (no longer in use) for AI interpretation. Interpretation was considered positive for OMI if the AI software reported OMI at any confidence level. Interpretation was only considered normal if the AI reported “Not OMI with high confidence”; all other interpretations were considered abnormal. The PMCardio application diagnoses 38 ECG abnormalities in addition to OMI; however, the Telegram interface only diagnoses “OMI” or “Not OMI”. Therefore, any abnormality other than OMI which might have been missed by the CCA was not compared with AI in this study.

### 2.4. Primary Outcome

The diagnostic performance of the AI algorithm for OMI from the 12-lead ECGs misinterpreted as normal by the CCA was the primarily analysis. The performance was evaluated through several data comparisons: (1) the percentage of the first-recorded ECGs that were diagnosed as normal by the CCA, but diagnosed as OMI by AI; (2) the percentage of all the ECGs misread as normal by the CCA, but correctly interpreted as OMI by AI; and (3) the percentage of the ECGs misread as normal by the CCA, but interpreted by AI as abnormal (defined as OMI with any confidence or not OMI with low- or mid-level confidence).

The secondary aim of this study is to describe the common features of the ECGs indicative of OMI, but missed by the CCA.

### 2.5. Statistical Analysis

Simple statistics were used, with percentages and proportions only. CCA and AI were compared with Chi-square. All the data were compiled in a spreadsheet program, and the program’s calculation functions were used to total the results.

## 3. Results

### 3.1. Participants

Sixty-five cases, with a total of 172 ECGs, were reviewed for inclusion. Six cases had ECGs manifesting OMI, which were read as normal by CCAs, but were ultimately excluded because they did not meet the inclusion criteria; five of the six had culprits with PCI, but had TIMI-3 flow without a very high peak troponin level. Thus, 59 cases met the inclusion criteria, with at least one 12-lead ECG recognized as OMI, but which was interpreted as “normal” or “otherwise normal” by the CCA, and the patient was ultimately diagnosed with OMI using the outcome definition. Seventeen cases were among the thousands of ECGs used to train the AI model; after excluding these cases, there were forty-two left for analysis. The blog posts reported the patient’s age in 31 (74%) cases and sex in 28 (67%) of cases. The average age was 53 years, old and 50% of the patients were male.

Of the 42 cases, angiography showed TIMI-0 flow in 26, TIMI-1 flow in 0, TIMI-2 flow in 4. Six had TIMI-3 or unspecified TIMI flow with a very high peak troponin level; six had TIMI-3 flow or unspecified TIMI flow, with a new wall motion abnormality, and an elevated troponin level diagnostic of acute MI, but either the peak troponin level was unknown or below the very high threshold.

### 3.2. CCA Interpretations of the Initial ECG

Of these 42 cases, the initial ECG was interpreted by the CCA as normal in 37 (88%); the exact diagnoses were as follows: (1) “Normal ECG” in 32 (86%) cases and (2) “Otherwise normal” in 5 (14%), with an additional qualifier of “sinus bradycardia” (n = 4, 11%) or “Marked sinus arrhythmia” (n = 1, 3%). Five patients (12%) had abnormal initial ECGs according to the CCA. The CCA interpretations among these five were STEMI (n = 2, 5%), a nonspecific ST-T wave abnormality (n = 2, 5%), and the right bundle branch block (n = 1, 2%). These five patients went on to have subsequent ECGs obtained in the ED prior to angiogram that were interpreted by the CCA as normal or otherwise normal in spite of manifesting OMI.

### 3.3. AI Interpretations of the Initial ECG

The AI software interpreted 34 (81%) of the 42 initial ECGs of each case as diagnostic of OMI. Of these interpretations, 32 (76%), 2 (5%), and 0 (0%) were made with high, medium, and low confidence ratings, respectively. Only six (14%) of the initial ECGs were interpreted by AI as normal (i.e., “Not OMI with high confidence”); the remaining thirty-six (86%) were interpreted as abnormal.

Of the 37 initial ECGs interpreted by the CCA as “normal” or “otherwise normal”, the AI interpreted 29 (78%) as OMI, 31 (84%) as abnormal, and 6 (16%) as normal. Among these cases, 27/29 (73%) were diagnosed as OMI with high confidence, 2 (5%) as OMI with mid-level confidence, 0 (0%) as OMI with low confidence, 1 (3%) as not OMI with low confidence, 1 (3%) as not OMI with mid-level confidence, and 6 (16%) as not OMI with high confidence.

### 3.4. Per-ECG Diagnostic Performance

A total of 96 ECGs were obtained prior to reperfusion therapy from the 42 patients included in this study; there were CCA interpretations available for 78 of these ECGs. The CCA interpreted 57 (73%) as normal or otherwise normal and 21 (27%) as abnormal. Specifically, forty-six (59%) ECGs were read as normal, seven (9%) as “sinus bradycardia but otherwise normal”, one (1%) as “early repolarization but otherwise normal”, one (1%) as “sinus tachycardia but otherwise normal”, one (1%) as “marked sinus arrhythmia, otherwise normal”, and one (1%) as “sinus rhythm with PVCs, otherwise normal”. The abnormal CCA interpretations included eight (10%) “STEMI”, two (3%) “moderate ST depression”, seven (9%) “nonspecific ST-T wave abnormality”, one (1%) “Anterior MI of indeterminate age”, three (4%) “Consider subendocardial injury”, one (1%) “possible left atrial enlargement”, one (1%) “prolonged QT”, two (3%) “Right bundle branch block”, and one (1%) “possible acute pericarditis” cases. CCA interpretation was unknown for 18 (19%) of the ECGs, but the STEMI criteria were satisfied for 5 of these 18, so their CCA interpretations were assumed to be “STEMI”.

**Figure 1:**
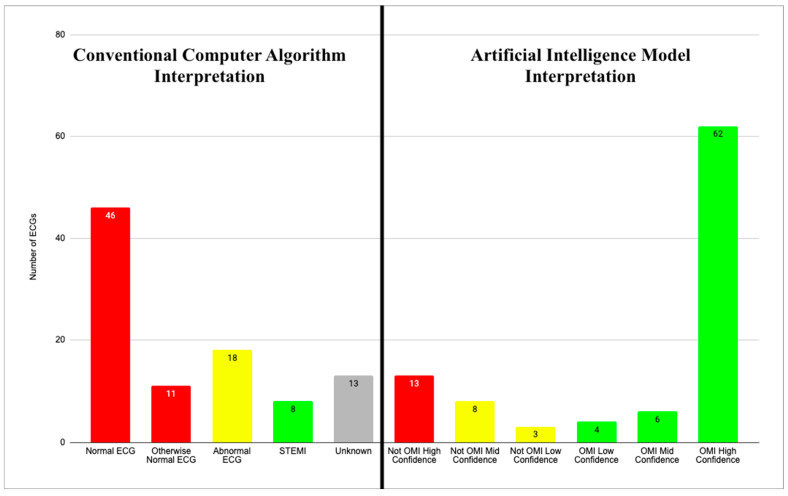
CCA and AI interpretations. Two graphs showing CCA interpretations for all 96 ECGs (**left**) and AI model interpretations for all 96 ECGs (**right**). Red bars represent normal ECG reads in both AI and CCA graphs. Yellow bars represent abnormal reads other than acute MI. Green bars represent ECGs with interpretation of acute MI. Gray bar represents unknown CCA reads.

The AI algorithm interpreted 72 of 96 (75%) ECGs as OMI, with 62 (65%), 6 (6%), and 4 (4%) with high, medium, and low confidence, respectively. AI interpreted 13 (14%) as normal (i.e., “Not OMI with high confidence”) and 83 (86%) as abnormal. This information is summarized in Figure 1 and Table 1.

### 3.5. AI Performance with CCA “Normal” ECGs

A total of 57 ECGs were read by the CCA as either “normal” or “otherwise normal”. Of these, the AI correctly interpreted 46 (81%) as abnormal and 41 (72%) as OMI. The abnormal reads by the AI included thirty-seven (65%) OMI with high confidence, three (5%) OMI with mid-level confidence, one (2%) OMI with low confidence, one (2%) not OMI with low confidence, and four (7%) OMI with mid-level confidence. The AI called 11 (19%) ECGs not OMI with high confidence.

### 3.6. CCA vs. AI, Case by Case

To evaluate the diagnostic performance of the AI against the CCA case by case, the 42 cases were split into four separate categories:

1. Cases in which both the CCA and the AI system diagnosed acute MI.
2. Cases in which the CCA did not diagnose acute MI, but the AI system did.
3. Cases in which neither the CCA nor the AI system diagnosed acute MI.
4. Cases in which the CCA diagnosed MI, but the AI system did not.

There were eight cases in which both the CCA and the AI system diagnosed acute MI. These cases are summarized in Table 2. In these cases, the AI identified acute MI a mean of 68.6 min faster than the CCA. The AI identified acute MI after a mean of 1.25 ECGs, whereas the CCA identified acute MI after a mean of 2.25 ECGs.

**Figure 2:**
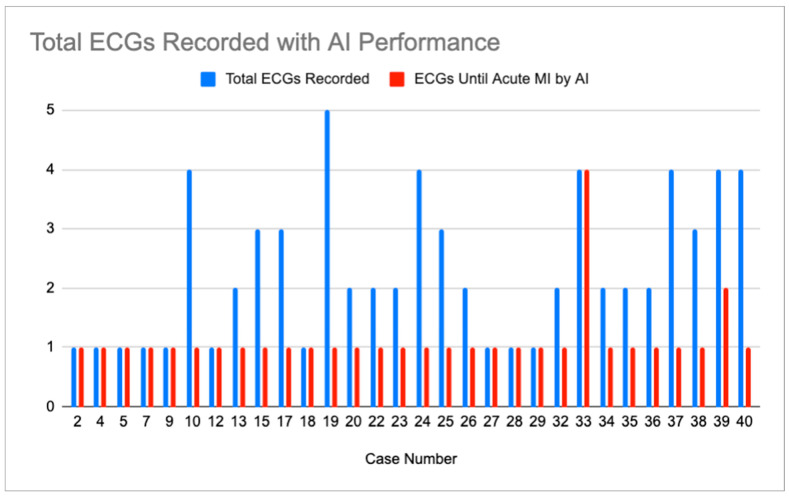
Display showing number of ECGs recorded in cases where CCA did not identify acute MI (blue bar) and number of ECGs recorded in each case until AI system recognized OMI at any confidence level (red bar).

There were 30 cases for which the CCA did not, but the AI did diagnose acute MI on any ECG. These cases include all the CCA reads of abnormal, except for “STEMI”. A mean of 2.3 ECGs was recorded in these cases. The AI diagnosis of OMI at any confidence level was made after a mean of 1.13 ECGs. This is summarized in Figure 2.

There were four cases in which neither the CCA nor the AI diagnosed acute MI. Only in cases six and sixteen did the AI call every ECG “normal”, that is, not OMI with high confidence, and in these cases, the CCA also called every ECG “normal” or “otherwise normal”. These cases and their ECG reads are shown in Table 3.

In none of the 42 cases did an ECG with the CCA interpretation of acute MI or abnormal ECG receive an AI diagnosis of “Not OMI with high confidence”.

#### Common ECG Features

**Figure 3:**
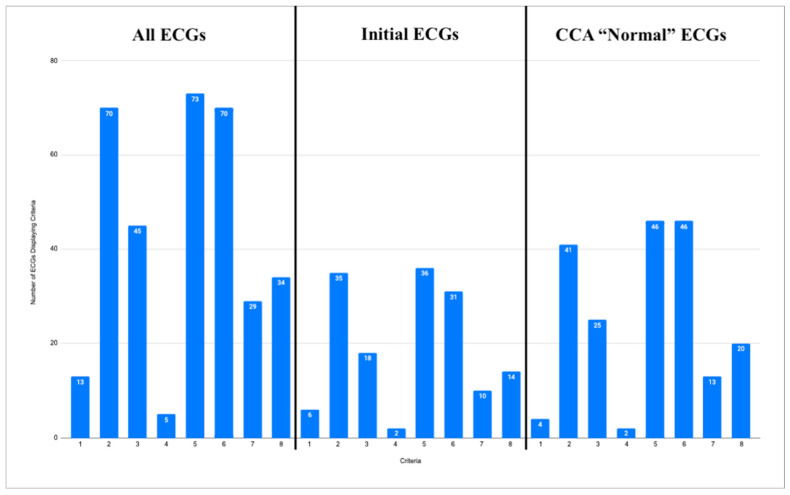
Three graphs showing frequencies of ischemic features described below for all ECGs (**left**), initial ECG of each case (**center**), and all ECGs called “normal” or “otherwise normal” by CCAs (**right**). Y axis shows number of ECGs for each graph. Graph labeled “All ECGs” accounts for all 96 ECGs from data set. Graph labeled “Initial ECGs” accounts for initial ECG of each of 42 cases. Graph labeled “CCA ‘Normal’ ECGs” accounts for all 57 ECGs in data set called “normal” or “otherwise normal” by CCAs. X axis enumerates 8 ischemic features for which each ECG was evaluated. Corresponding features are as follows: 1. STEMI criteria; 2. hyperacute T waves; 3. pathologic Q waves with ST elevation; 4. terminal QRS distortion; 5. reciprocal changes, including ST depression, T wave inversion, horizontal ST segment flattening, and down-up T waves; 6. subtle ST elevation; 7. any amount of ST depression in V1–V4; and 8. any inferior ST elevation with any reciprocal change.

**Figure 4:**
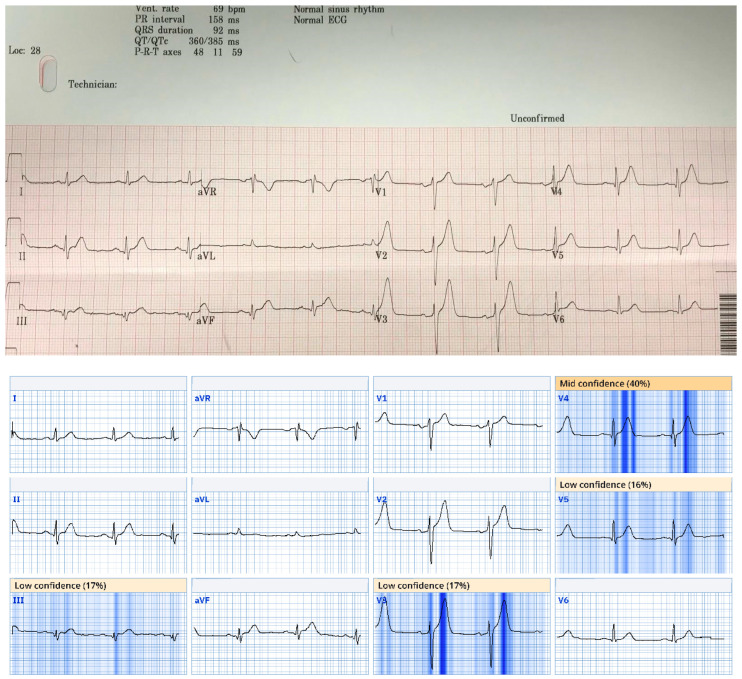
This ECG from case 5 was called normal by Marquette 12 SL algorithm; however, there are hyperacute T waves in anterior and lateral precordial leads and pathological Q waves with ST elevation and hyperacute T waves in inferior leads. Patient was found to have a 100% occlusion of proximal LAD. Queen of Hearts software calls this ECG “OMI with high confidence”. Digitized version shows AI explainability, in which parts of ECG most concerning for OMI to AI are highlighted in blue.

**Figure 5:**
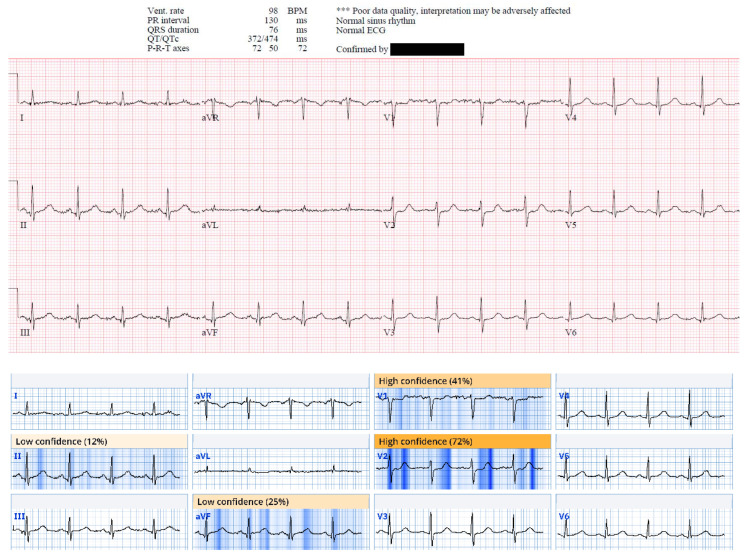
This ECG from case 19 was called normal by Marquette 12 SL algorithm; however, there is ST depression in V1 and V2 suggestive of acute posterior OMI, and there are small Q waves associated with subtle ST elevation in inferior leads, reciprocated by subtle T wave inversion in aVL. This patient was found to have 95% mid RCA culprit that was stented, peak troponin I of 1300 ng/L, and new basal inferior wall motion abnormality. Queen of Hearts calls this ECG “OMI with high confidence”. AI explainability map is shown below original tracing.

Of all 96 ECGs included in this study, the most common ischemic feature present was reciprocal change, including reciprocal ST depression, reciprocal T-wave inversion, reciprocal horizontal ST flattening, and reciprocal down-up T-waves in 73 (76%) ECGs. The next most common ischemic features were hyperacute T waves and subtle ST elevation not meeting the criteria for STEMI, each present in 70 (73%) of the tracings. Among the 42 first ECGs obtained in each case, reciprocal changes were most common, seen in 36 (86%) ECGs, followed by hyperacute T waves in 36 (83%) and subtle ST elevation in 31 (74%). Among the 57 ECGs labeled “Normal” or “Otherwise normal” by CCA, the most common ischemic findings were reciprocal changes and subtle ST elevation, each seen in 46 (81%) ECGs, followed by hyperacute T waves, seen in 41 (72%) ECGs. Curiously, there were four ECGs recorded which met the STEMI criteria, but received the diagnosis of “normal” by the CCA. Additional ischemic features and their frequencies are listed in Figure 3. See Figure 4 and Figure 5 for examples of the “normal” ECGs, which manifest OMI and were recognized by AI as OMI.

The ECGs meeting the criteria for STEMI were not counted as displaying subtle ST elevation. The ECG in case 7 could not be evaluated for the STEMI criteria, as lead V2 had 2 mm of ST elevation, and lead V3 only had 1.5 mm of ST elevation, and no patient demographics were given for this case. As such, 95 ECGs were evaluated for the STEMI criteria in the graph labeled “All ECGs”, 41 for the graph labeled “Initial ECGs”, and 56 for the graph labeled “CCA ‘Normal’ ECGs”.

**For Culprit Artery Analysis, see Table 4.** The left anterior descending (LAD) artery was involved in 18/42 (43%) cases; all 18 were diagnosed as OMI by AI. The left circumflex or its obtuse marginal branches were involved in 9/42 (22%).

## 4. Discussion

In the US, there are an estimated 10 million Emergency Department (ED) visits for chest pain per year [28]. All patients with chest pain undergo ECG recording primarily to rapidly diagnose acute coronary occlusion. Additionally, there are countless patients who present with less-specific symptoms of ischemia such as dyspnea, for whom ECGs are recorded for the same reason. All of these patients undergo one or more ECG recordings, amounting to at least 20 million ECGs. Acute coronary occlusion is typically diagnosed using ST elevation (STEMI), and CCAs are programmed for this purpose [29]. However, the guidelines recommended STE is present in less than 50% of acute OMI [30]. Findings beyond STE are more than twice as sensitive for OMI, but these findings are difficult for providers to learn [21].

There are approximately 800,000 myocardial infarctions (MIs) diagnosed per year in USA EDs [31]. Of these MIs, 300,000 are ST elevation MIs (STEMIs), and true positive STEMIs are well-known to represent acute coronary occlusion MIs (ACOMI, or OMI for short); 500,000 are Non-STEMIs, which are purported to represent the absence of acute total occlusion. However, because STE lacks sensitivity for OMI, 34% of Non-STEMI have an occluded artery with TIMI-0/1 flow on the next-day angiogram [32]. Thus, there are at least 170,000 OMIs which do not meet the STEMI criteria and can be classified as “subtle”. In other words, among 10 million initial ECGs recorded, approximately 1.5% are recorded in patients with subtle OMI, meaning that emergency physicians must view the scores of ECGs before seeing one with a subtle OMI, and furthermore most emergency providers are not equipped to make an accurate diagnosis on many of these often extremely subtle ECGs. Thus, providers rely on computer algorithms to detect whether the ECG is normal; abnormal, but non-diagnostic; or diagnostic of ischemia/infarction.

Most Non-STEMI ECGs that do not reveal overt ischemia receive a CCA diagnosis of “nonspecific” or “non-diagnostic” ST-T abnormalities rather than normal [33]. In a very large study published before the discovery of many subtle signs of OMI, 7.9% of all acute MIs (including both those with and without occlusion) had a “normal” ECG, and 35% had a “nonspecific” ECG [33]. The incidence of normal ECG in patients with active persistent OMI is unknown and doubtless much lower. In fact, a blinded expert interpreter was able to recognize OMI on the ECG in 91% of cases, with high specificity, as was the PMCardio Queen of Hearts AI model [16,21]. On the other hand, CCAs are only 62–69% sensitive for acute MI with a culprit [29].

In spite of the lack of sensitivity for OMI for a CCA “normal” ECG, several publications over the years have concluded that if the CCA states that the ECG is “normal”, the triage emergency physician need not review it, refs. [7,8,9,10], or that physician review of triage ECGs is not cost-effective [11]. However, it has previously been shown that the CCA interprets some OMI as completely normal [17], and these data support that.

In this retrospective case series, we examined the performance of an AI algorithm to detect OMI from ECGs previously interpreted as completely normal (in contrast to “nonspecific abnormalities”) by a CCA. These ECGs belonged to patients later determined to be suffering an acute coronary occlusion and were recognized by an expert as in ECG OMI recognition representing OMI; they were obtained both prehospital and in an ED prior to any reperfusion therapies. We found that AI interpreted 86% of the ECGs as abnormal and identified OMI in 75%. This finding was in contrast to the CCA which interpreted only 27% of all the ECGs as abnormal and identified acute MI in only 10%. A similar pattern was observed with regard to the first ECG obtained in each case, with AI interpreting 86% as abnormal and 81% as indicative of OMI. The CCA interpreted 12% of these initial ECGs as abnormal and just 5% as indicative of acute infarction. These data suggest the immense potential of AI for reducing false negative ECGs, both prehospital and in the ED. Additionally, these findings have implications for the triaging of patients with chest pain or potential ACS.

The PMCardio “Queen of Hearts” AI model employs a relatively new deep convolutional neural network technology which has been expert-trained and validated and is constructed on the OMI paradigm; it is the first algorithm of its kind, with double the sensitivity (for OMI) of that of the STEMI criteria at equal specificity [21]. It has also shown improved specificity over a conventional algorithm [34]. However, these are the first data published specifically focusing on this AI software’s ability to detect OMI that is so subtle that the conventional algorithm failed to find any abnormality.

The American College of Cardiology now specifies in the 2022 chest pain guidelines several ECG “STEMI Equivalents” beyond the STEMI criteria, warranting emergent angiography and intervention: posterior OMI, Sgarbossa, and Smith-modified Sgarbossa criteria for LBBB or ventricular paced rhythms, hyperacute T-waves, and DeWinter’s Sign [1]. This growing emphasis on “STEMI equivalents” is a shift away from the traditional STEMI-NSTEMI paradigm and toward the OMI-NOMI paradigm [2,4].

It is widely recognized that treating STEMI rapidly improves outcomes, but there is also support for the rapid treatment of STEMI-equivalents; in one study, for patients with either STEMI or equivalent, the mortality rate was 1.8–4.3% if treated within the guideline timing and 4.7–14.2% when the treatment time was longer [6]. Rapid treatment requires rapid detection, and this requires accurate ECG interpretation. It is axiomatic that if, in the case of OMI, the ECG is read by the computer as “normal” and that interpretation is trusted, that there will be diagnostic and treatment delays.

These results suggest that treatment times could be significantly shortened by the use of this software at triage. The AI model was consistently able to identify OMI ECG patterns on multiple ECGs and several hours before the CCA in some cases. In 88% of the cases of OMI, the initial ECG was interpreted by the CCA as normal when it, in fact, manifested OMI; trusting such an interpretation would lead to a delay in workup, while infarction is ongoing. On the other hand, the AI interpretation would have properly identified 81% of such cases as OMI and alerted the triage staff that the ECG was abnormal in 86% of cases. Thus, there is clear potential of this AI software to reduce the number of cases of missed OMI in the triaging of patients with ACS.

It is often stated that the artery most often involved in OMI that is not evident on the ECG is the circumflex. Here, we find that it was the anterior descending artery OMI, was the artery with the most CCA “normal” ECGs.

Finally, in these CCA “normal” ECGs which manifested OMI, we identified common important features that providers can look for, especially in patients with a high index of suspicion for acute MI. The three most common features were subtle STE, reciprocal changes (including ST depression, T-wave inversion, horizontal ST flattening, or down-up T-waves), and hyperacute T-waves.

## 5. Limitations

First, this is a study of a very select group of patients who were recognized to have OMI on an ECG and found to have an outcome of OMI, but who had one or more pre-angiography ECGs that manifest OMI, but were diagnosed as “normal” by the CCA. The data set is derived from a blog where ECGs are deemed interesting enough for publication. The blog posts may not represent the entirety of patients encountered or contain every ECG recorded during these encounters. Second, with this study design, no conclusions can be drawn regarding the rate of false positives when employing AI interpretation. However, it is known that the AI model does not overdiagnose OMI. First, it was shown that the model has twice the sensitivity of the STEMI criteria at a fixed specificity of 97.7% [21]. Moreover, although the previous studies have demonstrated a false positive rate of 10–36% for the diagnosis of STEMI in the ED [35,36,37,38], in a large retrospective study of STEMI activations in the Midwest STEMI Consortium, the AI model accurately identified many false positives, decreasing the false positive rate from 25% to 16% [39]. In a retrospective prehospital study, the AI model would decrease the OMI false positives from 59% to 29% [34]. However, the present study addresses a different specificity question, and that is whether the AI model will overcall “abnormal” (i.e., “Not OMI with High Confidence”); this is unknown, and future studies are required in order to ascertain this. Finally, if one were to object that an AI interpretation of “Not OMI with mid-level confidence” should also be classified as “normal”, then of the thirty-seven initial ECGs interpreted by the CCA as normal, AI would have diagnosed nine, rather than six as normal; among the twenty-nine OMIs that were diagnosed as normal by the CCA, AI would have diagnosed evesn, rather than six as abnormal.

## 6. Conclusions

The Conventional Computer Algorithm (CCA) may interpret ECGs that are diagnostic of OMI as “normal”. The PMCardio “Queen of Hearts” AI OMI ECG model identified the vast majority as OMI, and even more as abnormal.

## Figures and Tables

**Table 1:** CCA interpretations of “otherwise normal” or “abnormal”, with associated specific interpretations.

Otherwise Normal ECG Reads	Total	Abnormal ECG Reads	Total
Sinus Bradycardia	7	Nonspecific ST-T Wave Abnormality	7
Sinus Tachycardia	1	Consider Subendocardial Injury	3
Marked Sinus Arrhythmia	1	Moderate ST Depression	2
Frequent PVCs	1	Right Bundle Branch Block	2
Early Repolarization	1	Anterior MI of Indeterminate Age	1
-	-	Possible Acute Pericarditis	1
-	-	Possible Left Atrial Enlargement	1
-	-	Prolonged QT	1
Total “Otherwise Normal”	11	Total “Abnormal”	18

**Table 2:** Comparison of ECG interpretations for cases in which both CCA and AI system recognized acute MI. If available in blog post, specific CCA is named in “Interpretation Software” column. For each case, once software identifies acute MI, that is, once CCA calls STEMI or AI calls OMI at any confidence level, ECGs in corresponding row are no longer enumerated. Time differences between ECGs are displayed in minutes. Cases 11 and 30 are included because later ECGs which manifested OMI were interpreted as normal by CCA.

Case Number	Interpretation Software	ECG 1	ECG 2	ECG 3	ECG 4
1	Unknown CCA	Normal	Unknown	STEMI (Assumed)	
	QoH	OMI Mid		(+150 Min)	
3	Unknown CCA	Normal	Unknown	STEMI (Assumed)	
	QoH	Not OMI High	OMI High	(+40 Min)	
11	Zoll Algorithm	STEMI (Assumed)			
	QoH	OMI High			
14	Marquette 12 SL	Normal	Normal	Abnormal	STEMI
	QoH	Not OMI High	OMI High		(+120 Min)
21	Unknown CCA	Normal	STEMI (Assumed)		
	QoH	OMI High	(Unknown time)		
30	Marquette 12 SL	STEMI			
	QoH	OMI High			
41	Marquette 12 SL	Normal	STEMI		
	QoH	OMI High	(+125 Min)		
42	Marquette 12 SL	Normal	STEMI (Assumed)		
	QoH	OMI High	(+45 Min)		

**Table 3:** Comparison of ECG reads for cases in which neither CCA nor AI system identified acute MI. If available in blog post, specific CCA is named in “Interpretation Software” column.

Case Number	Interpretation Software	ECG 1	ECG 2	ECG 3
6	Unknown CCA	Normal		
	QoH	Not OMI Mid		
8	Marquette 12 SL	Otherwise Normal		
	QoH	Not OMI Mid		
16	Marquette 12 SL	Normal	Otherwise Normal	Normal
	QoH	Not OMI High	Not OMI High	Not OMI High
31	Unknown CCA	Normal		
	QoH	Nor OMI Low		

**Table 4:** This table represents frequency of each culprit artery for cases included in this study. Left anterior descending artery was most common culprit, followed by right coronary artery. Total is 46, rather than 42 because cases 12, 26, 34, and 39 involved two culprit arteries, both of which are accounted for in this table.

Culprit Artery	Number of Cases
LAD	18
Diagonal	4
Circumflex	2
Obtuse Marginal	7
RCA	12
PDA	2
Ramus	1

## Data Availability

The data presented in this study are not available on request from the corresponding author due to privacy or ethical restrictions.
